# Functional Endophytes Regulating Plant Secondary Metabolism: Current Status, Prospects and Applications

**DOI:** 10.3390/ijms24021153

**Published:** 2023-01-06

**Authors:** Zhaogao Li, Keyi Xiong, Weie Wen, Lin Li, Delin Xu

**Affiliations:** 1 Department of Cell Biology, Zunyi Medical University, Zunyi 563099, China; 2 Engineering Research Center of Key Technology Development for Guizhou Provincial Dendrobium Nobile Industry, Zunyi Medical University, Zunyi 563099, China

**Keywords:** endophytes, biosynthesis mechanism, secondary metabolites, SynComs, metaomics

## Abstract

Endophytes, which are widely found in host plants and have no harmful effects, are a vital biological resource. Plant endophytes promote plant growth and enhance plants’ resistance to diseases, pests, and environmental stresses. In addition, they enhance the synthesis of important secondary metabolites in plants and improve the potential applicability of plants in agriculture, medicine, food, and horticulture. In this review, we summarize the recent progress in understanding the interaction between endophytes and plants and summarize the construction of synthetic microbial communities (SynComs) and metaomics analysis of the interaction between endophytes and plants. The application and development prospects of endophytes in agriculture, medicine, and other industries are also discussed to provide a reference for further study of the interaction between endophytes and plants and further development and utilization of endophytes.

## 1. Introduction

Endophytes are diverse and valuable resources in nature with the potential for human exploitation. In the early stage of endophyte discovery, the soil microbiota was the main seed bank for bioactive compounds [1,2]. In recent years, the search for microbial candidate sources with high application potential has become an area of increasing focus, as the repeated discovery of known metabolites from soil microorganisms has become more frequent. There is a great deal of interest in the potential of microorganisms from poorly studied environments, including plant-related microorganisms such as endophytes. Undoubtedly, as essential components of the plant microecosystem, endophytes have a profound influence on the synthesis and accumulation of various secondary metabolites in the host plant. To date, active substances with significant therapeutic effects, such as anticancer drugs, antibiotics, antiviral drugs, antidiabetic drugs, and immunosuppressive compounds, have been isolated from poorly studied endophytes interacting with plants [3,4,5,6]. Despite some advances, the molecular mechanisms by which endophytes influence the occurrence of metabolic substances in plants are still unknown. Fortunately, further analysis of the biological features, structure–activity relationships, and modes of action between endophytes and plants can aid in the further investigation of their interrelationships. This will facilitate the exploration of the molecular mechanisms and related signaling pathways via which endophytes affect the generation of plant secondary metabolites [7].

Secondary metabolites (SMs) are the products formed by interactions with the environment during plant growth and development. SMs mainly include alkaloids, flavonoids, terpenoids, peptides, phenols, sterols, and additional minor molecular organic compounds [8,9]. As essential substances used by plants for self-protection to cope with their environment, SMs have various physiological functions, such as regulating plant growth and biological defense, and they are also involved in the plant response abiotic stresses, such as drought, low temperature, salinity, and metals [10,11,12,13]. In recent years, research on plant SMs has developed rapidly, especially in of the fields of human health and agricultural production [14]. However, the yield of SMs from plants is very limited and is far from meeting the growing market demand. Therefore, it is imperative to find ways to improve the biosynthesis of SMs in plants.

To date, several reviews on plant SMs have been published [15,16,17], but there are few systematic reviews on how endophytes affect the synthesis of plant SMs [18,19,20]. In this review, we have summarized the development of endophyte–plant interactions and the molecular mechanisms of endophyte–plant SM interactions, and we have presented a platform for endophyte–plant interactions. Furthermore, the possible role of endophyte-promoted plant SMs in plant growth induction, structure–activity relationships, mechanisms of action of SMs in plants, and practical applications in human life are discussed.

## 2. Basis of the Interaction between Endophytes and Plants

Endophytes play a significant role in promoting the growth of host plants, and conversely, plant bodies are also crucial for the adaptation of endophytes to complex environments [21]. An essential manifestation of the host plant’s influence on endophytes is that it provides an environment with sufficient water and nutrients for endophyte colonization, either inside or on its surface, and forms certain special structures [22]. Endophytes are frequently observed in plants in the form of multicellular aggregate communities, which have attracted significant attention in recent years because they can be regarded as a special “microecological” system formed by endophytes and host plants [23,24,25]. In this system, a series of distinct interactions occur between endophytes and plants. These interactions help endophytes resist environmental changes more effectively and assist plants in producing biological products conducive to their growth.

### 2.1. Evolution of Interactions between Endophytes and Groups of Plants

**Figure 1:**
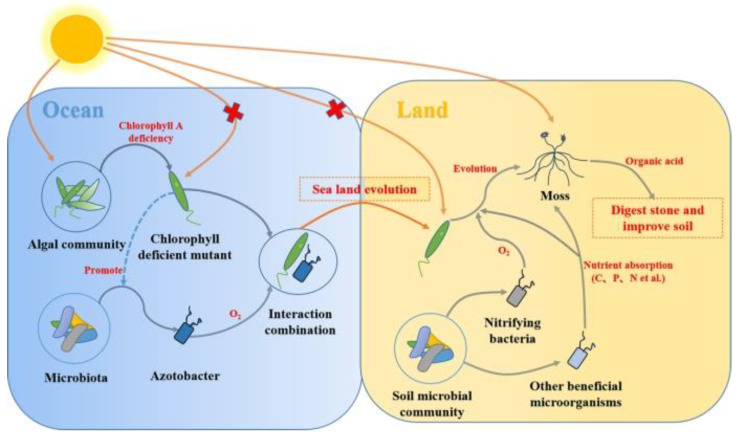
Model diagram of endophytes promoting the evolution of lower plants from aquatic to terrestrial conditions. In the ocean, the evolution of chlorophyll-a made it possible for lower algae to obtain O_2_ by forming interactions with nitrogen-fixing bacteria and then migrate onto land. On land, nitrifying bacteria and other beneficial microorganisms can obtain O_2_ and nutrients needed to maintain their own growth, resist environmental stress, and evolve into shade-loving mosses. Finally, organic acids produced by mosses digest stones and fertilize the soil.

Endophytes interact with plants over extended periods of time. In nature, the ocean is the cradle of life, with algae and bacteria being the main remaining members from the early ocean. They produce oxygen during metabolism to support life and promote the switch from aquatic to terrestrial life [26,27]. Two significant factors influence the transition from aquatic to terrestrial life in plants (Figure 1). In the absence of chlorophyll molecules, plants can survive on land by reducing their light-catching surface, which is also suitable for the dark habitat of early terrestrial bryophytes [28]. In addition, the early interaction with soil microorganisms enabled moss to survive on land for a long time, which was the beginning of the interaction between plants and microorganisms in evolutionary terms [29]. Fossil evidence from 400 million years ago strongly suggests that plants engaged in symbiotic associations with mycorrhizal fungi [30], and the adaptive value of this ability has been preserved throughout plant evolutionary history. However, reliable evidence of interactions between plants and microorganisms found in modern studies came from the 1930s: when livestock ate endophytic fungus-infected herbage, great losses occurred in the animal husbandry industry, leading to the initial understanding of endophytic bacteria in plants [31].

A major problem facing humanity in the 21st century is the lack of plant resources [32], a challenge further exacerbated by human population growth, soil degradation, and pollution from various environmental factors, including floods, droughts [33], salinity [34], temperature (extreme heat and cold) [35], and heavy metals [36]. Plants are saprobic, but they have evolved defense mechanisms for sensing and adapting to stressful environments over long periods of evolution. Some common and extensively used methods include osmotic agent production, altered water movement, and scavenging of reactive oxygen species [32]. Endophytes, as an essential link in the adaptation of plants to environmental changes, undoubtedly play an essential role. Indeed, as endophytes are being intensively studied, their interaction with plants has great potential value for effectively addressing the current shortage of plant resources. Therefore, it is essential to explore the evolution of endophyte–plant interactions.

### 2.2. Changes in the Genetic Material of Endophytes Interacting with Plants

**Figure 2:**
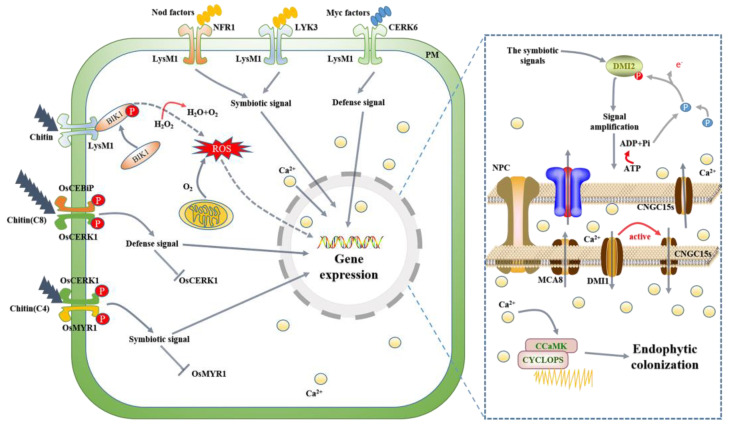
Patterns of molecular mechanisms underlying the initiation of symbiotic or immune responses of plants to signaling molecules during mycorrhizal interactions. LysM1 receptor modules interact with different proteins to form unique-acting receptors on plant cell membrane surfaces (NFR1, LYK3, CERK6, OsMYR1-OsCERK1, OsMYR1-OsCEBIP et al.), and some receptors antagonize each other, such as the OsMYR1-OsCERK1 receptor and the OsMYR1-OsCEBIP receptor. When mycorrhizal interactions occur, extracellular signal molecules (Nod factor, Myc factor, chitin et al.) with these particular receptor combinations form defense or symbiotic signals in the cytoplasm, which initiate the plant cell’s internal defense or the expression of related genes. In addition, symbiosis and defense reactions in the process of symbiosis are accompanied by strong calcium oscillation in the nuclei. In calcium excitation, the symbiotic signal activates DMI2 protein under the action of related receptors, and the related channel proteins DMI1 and CNGC15s on the nuclear membrane are activated through the signal cascade reaction. The electrochemical signal in the nucleus changes dramatically, resulting in the movement of Ca^2+^ between the inner and outer membrane of the nucleus and the perinuclear lumen.

Plant genes are fundamental to cellular processes and defense mechanisms in general. However, long ago, plants actively evolved genetic modules to support symbiosis and benefit from the presence of microorganisms. This represented an essential genetic shift in plants from aquatic to terrestrial life and formed a strong basis for active genetic innovation in plants. Despite the risk of invasion by pathogens, these modules are still retained in plants and support the interaction between plants and endophytes [37]. Currently, the mechanisms by which plants respond to invading microorganisms by symbiosis or immune response are unknown, but some exciting discoveries have been made (Figure 2). An intriguing example involves a LysM receptor involved in mycorrhizal interactions. Nearly all terrestrial plants have LysM receptors that ensure the detection of various microbial signals. LysM receptors have a variety of minor molecular phantom structures on their surface that can bind to different proteins to form specific receptors. Chitin on the surface of pathogens or symbiotic signaling molecules (Nod factor, Myc factor) produced by beneficial microorganisms can be efficiently recognized, and the output of signals for the antimicrobial defense of plants against pathogens or the symbiotic reaction between microbes and plants can be controlled [38,39]. During this process, intense calcium ion shock events occur in the nucleus in response to the incoming symbiotic signal. In addition, during mycorrhizal symbiosis, OsMYR1, as a fungal receptor, can accurately identify the differences in chitin between fungi and recruit beneficial endophytic fungi [40].

Changes in genetic material are important for plant evolution and the core factor affecting endophyte–plant interactions. The interactions of early microbes depended only on the shallow surface area or on free space in the plant body [29,41], and these interactions did not lead to the formation of a symbiotic association in the traditional sense. Deep symbiosis later resulted from horizontal gene transfer (HGT) between species. Compared with vertical gene transfer (VGT), the greatest innovation of HGT is that it disrupts the reproductive isolation between species and makes the communication of genetic material between various organisms in nature more complex and diverse [42,43]. At present, HGT is known to commonly occur in prokaryotes and unicellular eukaryotes, but in multicellular eukaryotes, HGT is often considered to occur less frequently [44]. As observed in plants with endogenous bacteria, gene transfer in multicellular eukaryotes is a landmark event. On the one hand, these transfer events increase the genetic diversity of organisms, expanding the horizons of scientific research. On the other hand, they give plants and endogenous bacteria the ability to adapt to their changing environment, as plant–endophyte interactions are very important for genetic stability [45]. Therefore, the identification of changes in genetic material between plants and endophytes may allow us to quickly reveal the mechanisms related to microecological system balance and function during interactions between plants and endophytes.

### 2.3. Progress in Research on Endogenous Microecosystems

Plants actively seek the help of microbes to survive stressful situations. Over the past decade, there has been a paradigm shift in plant science, with the discovery of microbial functions and community composition increasingly being seen as drivers for improving the functioning of plant hosts. The endophytic microbiome can expand the plant genome and metabolic capacity to provide or promote a range of functions that help plants maintain basic life activities, including nutrient acquisition, immune regulation, and biological stress tolerance. Although endophytic microbial communities have been proposed as the next platform for the Green Revolution, basic research on the mechanisms of microbial community assembly and activity is still in its infancy [46]. Fortunately, in recent years, some progress has been made in the construction of a microecological balance between endophytes and plant hosts. Based on these findings, it is possible to decipher the functional diversity and complexity of the spatiotemporal dynamics of plant microbial communities and explore the balance of the endophyte microecological system [47].

Few topics in plant biology have aroused as much interest and controversy as endophyte–plant microecosystems. However, although the phenomenon of endophyte–plant microecosystems is now widely accepted, there are still various questions about how and why endophytes develop, how they maintain balance, and how they affect the growth processes of plants [25]. Numerous research results show that when plants recruit beneficial microbes, the symbiotic effects can increase the release of key molecular compounds during endophyte colonization [48,49]. Some plants can even regulate the absorption of nutrients and the expression of related genes at different stages of successful endophytic colonization. Endophytes that successfully colonize plants quickly use plant bodies to obtain the nutrients they need and rapidly form a stable collection of microbial communities, also known as the microbiome [50]. The microbiome is present in all parts and tissues of plants, forming a microecosystem with comprehensive linkages that can help all organisms cope with the overall impact of environmental conditions on the microecosystem and provide benefits to both microbial communities and plants.

**Figure 3:**
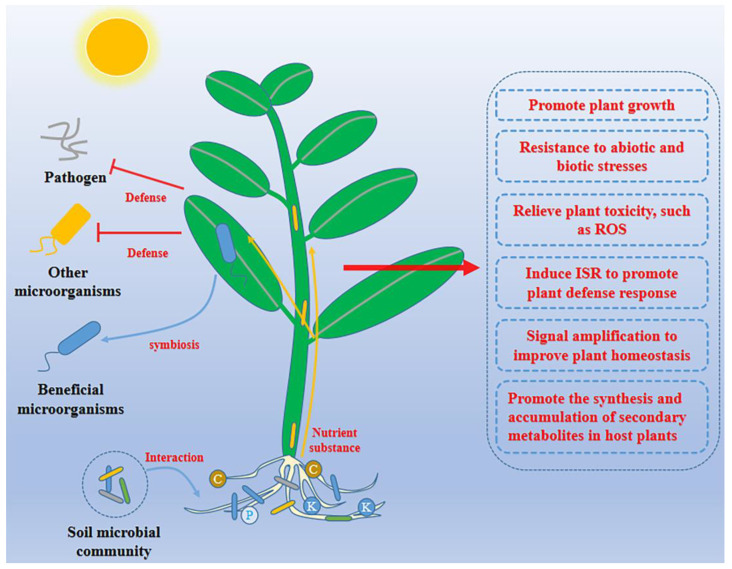
Plants recruit beneficial microorganisms to colonize and maintain the stability of the microecological system. As the plant grows, soil microorganisms are enriched, forming soil microbial communities at the roots. On the one hand, the interaction of microorganisms is used to improve the living environment, and on the other hand, inorganic elements in the soil (C, P, K, et al.) are enriched to provide nutrients needed for growth and development. In this process, plants release special substances that attract certain microorganisms from the environment and direct the colonization of beneficial microbes, triggering a range of physiological and biochemical reactions.

To date, many unique metabolic substances have been discovered in plant–endophyte microecosystems. These substances provide a reference for successfully revealing the types of interactions in endophyte–plant microecosystems. For example, microorganisms can stimulate plant growth by metabolizing tryptophan and other small molecules in plant secretions and producing plant hormones, including coenzymes, gibberellic acid (GA), cytokinin (CTK), and plant hormone analogues [51]. Coenzymes can also induce the transcription of 1-aminocyclopropane-1-carboxylate (ACC) synthetase and catalyze the formation of ACC. As the direct precursor of ethylene, ACC can be metabolized by endophytes through ACC deaminase to ameliorate abiotic stress [52]. Plant-associated microbiome members also produce a series of enzymes that detoxify reactive oxygen species (ROS), thereby minimizing plant-induced stress. In addition, the plant-associated microbiome protects plants from pathogens by producing antibiotics, lyases, and volatiles. Various microbial structures, such as secretory systems, flagella, and cilia, and proteins, including effector proteins, indirectly contribute to plant defense responses by triggering the induced systemic resistance response (ISR) [53]. At the same time, the microbial populations involved in the interactions within and between species also maintain a balance in the ecosystem to protect plants from pathogens, and the core endogenous bacteria can also amplify signals from the host, promoting microbiome restructuring to form the appropriate community structure in time and space under dynamic conditions to provide benefits to the plant [54]. Endophytes can alter the formation of secondary metabolites in plants, and they also produce many unique metabolites to enrich the resource pool of plants. In general, the stable formation of endophytes and plant microecosystems improves the growth performance and health status of plants and plays an increasingly important role in the process of plant growth and development (Figure 3).

## 3. Molecular Mechanisms Affecting the Secondary Metabolites of Endophytes in Plants

Endophyte action is a popular research topic in research on plant secondary metabolite formation and its influencing factors. Previous studies have suggested that the synthesis of diverse secondary metabolites in plants is related to the influence of various environmental factors, such as temperature, light, and microorganisms. Endophytes, as important environmental factors, play an important role in the formation and accumulation of secondary metabolites in plants. There have been many studies on the mechanisms by which endophytes promote the synthesis of secondary metabolites in plants, but there is no unified view within the scientific community. At present, there are four main hypothesized mechanisms: endophytes promote the accumulation of photosynthetic material in plants [55,56,57,58]. Endophytes regulate the expression of genes related to plant secondary metabolites [59,60,61]. The transfer of endophytes leads to the formation of compounds or alters the genetic material within plants [62,63]. The synthesis of unique secondary metabolites by endophytes affects plant material synthesis pathways [57,64].

### 3.1. Endophytes Promote the Accumulation of Photosynthetic Material in Plants

Endophytes promote the accumulation of photosynthesis-related substances, increase plant nutrient intake, and induce the synthesis of secondary metabolites. More than 90% of plant dry matter comes from the carbon fixation reaction of photosynthesis, and the organic matter assimilated by photosynthesis is the material basis for the formation of plant metabolites. Therefore, improving the efficiency of the utilization of light energy by plants is an important way to improve crop yield [65]. As reliable partners of plants, endophytes play an important role in improving the photosynthetic efficiency of plants [66] (Table 1). It was found that the water utilization rate of rice inoculated with endophytic fungi increased significantly and was positively correlated with daylight integration [61]. In addition, when nicotinamide adenine dinucleotide phosphate (NADPH)-dependent dehydrogenase was introduced into cyanobacteria, cell growth was significantly accelerated, photosynthetic efficiency was increased by approximately 50%, and cell activity increased. It was further found that the light saturation point of the modified cyanobacteria doubled, indicating that they could tolerate higher light intensity, which is important for adapting to drastic changes in light intensity in nature [67]. Therefore, we speculate that when an endophyte forms a symbiotic relationship with a plant, the endophyte changes the absorption of external substances, such as water molecules and inorganic salts, by the plant. Additionally, when the plant body is enriched with nutrients, the endophyte rapidly uses the required substances to form a large amount of NADPH and adenosine triphosphate (ATP) to supply photosynthetic material to the plant body, which improves the photosynthetic efficiency of the plant body, accelerates the enrichment of nutrients needed for growth, and thus improves the rapid accumulation of secondary metabolites.

### 3.2. Endophytes Affect the Expression of Genes Related to Plant Secondary Metabolites

Endophytes regulate genes related to the synthesis of secondary metabolites by plant cells through their signal transduction pathways. Endophytes also change the rate and intensity of the expression of these genes and induce the production and accumulation of corresponding secondary metabolites in plants (Table 2). The results showed that the levels of nutrients (N, P, K, Ca, Mg, Mn, Fe, Cu, and Zn) and related quality parameters (polysaccharide, total soluble protein, tea polyphenols, catechin, total flavonoids, and theobromine) were significantly increased by inoculating tea plants with arbuscular mycorrhizal fungi (AMF). In addition, the enzyme-encoding genes *CsAPX*, *CsTCS1*, *CsPAL*, *CsC4H*, *CsF3H*, and *CsDFR* showed the same trend as their corresponding quality parameters, indicating that AMF could promote the synthesis of secondary metabolites by upregulating the expression of related genes, thus affecting tea quality [42]. Other results showed that the culture biomass of *Euphorbia officinalis* was increased by 19.35% after treatment with an elicitor from the endophytic fungus *Fusarium oxysporum*, while the levels of isoflavin and diphenol were 5.81 times and 3.56 times those in the control, respectively. The activity of 1-deoxy-D-xylosulfos-5-phosphate synthetase (the key enzyme for the biosynthesis of isofluoromycin) was not significantly improved, while the activity of 3-hydroxy-3-methylglutaryl coenzyme A reductase (the key enzyme for phenol) was 1.67 times higher than that in the control [60]. In addition, the total levels of nine monomeric saponins in 1- to 4-year-old ginseng treated with Bacillus polymyxis spraying and root irrigation were 36.83%, 44.52%, 67.96%, and 79.44% higher than those in the control from the same year. At the same time, coculture of *B. polymyxis* and ginseng significantly increased the levels of 12 kinds of ginsenoside monomers, especially the levels of the rare ginsenosides CK and protopanaxadiol, which increased by 1.38- and 7.78-fold, respectively [80,81].

### 3.3. Compound Formation and Genetic Material Transfer in Endophytes and Plants

Transfer of compounds or genetic material occurs between endophytes and hosts (Table 3). *Phomopsis* sp., an endophytic fungus isolated from orchids in northern Ecuador, secretes the volatile organic compounds junienes, which are found only in higher plants [89]. The roots of liquorice plants colonized with *Bacillus pumilus* showed increased levels of total flavonoids, total polysaccharides, and glycyrrhizic acid. After inoculation with *B. pumilus*, the expression levels of *HMGR*, *SQS*, and *β-AS*, the key enzymes of glycyrrhizin synthesis, were significantly increased. These results indicated that *B. pumilus* could promote the growth of Ural hay by modifying the accumulation of antioxidants and increasing the glycyrrhizin content by changing the expression of key enzymes [90]. In addition, a large number of bacteria, such as *Lysinibacillus fusiformis*, *Bacillus megaterium*, *B. lichenifera*, *B. pumilus*, *Brevibacterium halotolerans*, *Achromobacter xylosoxidans* and *Pseudomonas putida*, were isolated from the roots of the halophyte *Prosopis strombulifera* in the Khewra salt region of Pakistan. The plant hormone abscisic acid (ABA) was widely present in these isolates [91]. Researchers isolated the strain *Kocuria turfanensis* 2M4 from the rhizosphere of salt-tolerant plants. This strain exhibited IAA production, phosphorus solubilization, and ferrophilia. In conclusion, the same or similar secondary metabolites were produced when endophytes interacted with plants, which suggested that compounds and even genetic material might be transferred between them during symbiosis.

### 3.4. Synthesis of Unique Secondary Metabolites by Endophytes Affects Plant Material Synthesis Pathways

Endophytes can synthesize compounds that the host cannot synthesize, or can only synthesize small amounts, changing the method of metabolite synthesis in the host and affecting metabolite levels. *Sphingomonas melonis*, an endophytic bacterium found in rice, confers resistance to disease-prone phenotypes by producing anthranilic acid. When *Burkholderia plantarii* infects rice, *S. melonis* secretes the small extracellular signaling molecule ammonia acetic acid to coordinate the host response and then interferes with the regulation of the *RpoS* transcriptional cascade that is dependent on the virulence factor biosynthesis pathway of *B. plantarii*. This prevents *B. plantarii* infection and promotes the accumulation of plant secondary metabolites [103]. By comparing the volatile substances of mint seedlings without fungi and those infected with endophytic fungi, significant differences in the components of mint roots, stems, and leaves, and even new compounds were found in mint infected with fungi [63]. In addition, Peng [104] screened an endophytic fungus capable of transforming curcumin from turmeric rhizomes. The conversion products were demethylcurcumin and dimethylcurcumin. Demethylcurcumin has higher antioxidant and anti-inflammatory activities than curcumin. Fu [105] screened four endophytes capable of transforming ursolic acid from the stems and leaves of the medicinal plant *Huperzia serrata*. These endophytes enhanced the production of ursolic acid. Denise [106] used an endophytic bacterium of the genus Anthracis to oxidize betulinic acid and transform it. This oxidation reaction is similar to the metabolic pathway of betulinic acid in mammals, providing a new method for studying this metabolic pathway in plants.

## 4. Progress in Research on Endophytes Acting on Secondary Metabolites in Plants

### 4.1. Construction of the SynComs Model for Endophyte–Plant Interactions

**Figure 4:**
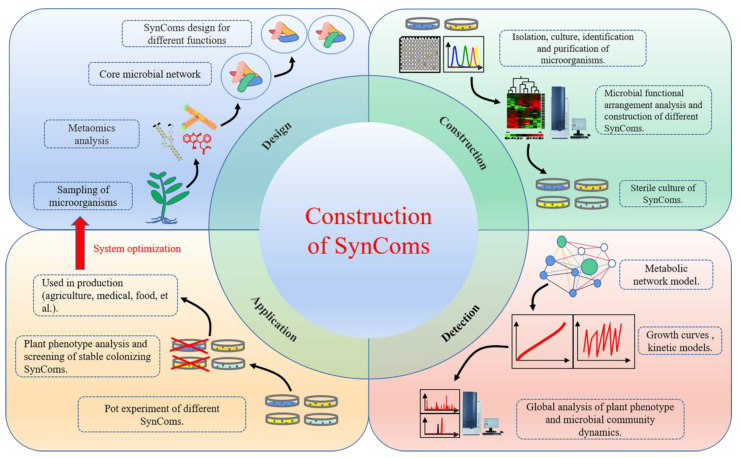
Construction of SynComs. SynComs with specific functions are formed by the recombination of microorganisms with different functions, depending on the needs of the plant. Functional analysis of the recombinant microbial community was performed using an omics approach. Core microbial communities that can play a stable and efficient role in plant growth and development, synthesis, and accumulation of secondary metabolites can be screened for commercial mass production. The function and composition of the SynComs were also optimized through practice.

Microorganisms rarely exist alone, and there is a positive correlation between species diversity and community productivity in nature. The ubiquity of these microbial communities in nature highlights the possible advantages of endophytic strains in coculture [107]. However, the study of plant secondary metabolites relying on only the naturally formed endophyte community is becoming increasingly limited. Researchers have gradually shifted their focus to identifying methods to better study the occurrence and accumulation of secondary metabolites under the interaction between plants and endophytes. SynComs is a process of artificially mixing different microorganisms with distinct species and functions in certain proportions under certain conditions to create a stable microbial community with distinct functions. With the development and implementation of SynComs, research on plants and endophytes advanced further (Figure 4). SynComs consider multiple types of interactions and the functions of different microorganisms under certain conditions to determine the proportion of mixed, stable, and functional microbial communities that can promote plant growth, nutrient uptake, and stress resistance. These interactions play an important role in such aspects of secondary metabolite production and can be applied to industrial production.

SynComs were first reported in the early 21st century. *Saccharomyces cerevisiae* was genetically modified through genetic hybridization, and two nonmating strains, R and Y, with different metabolic abilities were obtained. R synthesized lysine but needed adenine for growth, and Y synthesized adenine but needed lysine for growth [108]. A stable and sustained cooperative relationship was formed after coculture of the two strains, which laid a foundation for the development of SynComs in the future. Further studies showed that internal and external factors played an important role in the stability of the SynComs [109,110,111,112]. In 2014, researchers developed the concept of SynComs and defined SynComs as a coculture system established by two or more microorganisms in a substrate with a clear composition [113]. Subsequently, the importance of host genotypes and core microorganisms for the construction of SynComs was further revealed [114,115,116]. The above research results greatly promoted the development of SynComs technology and laid a solid foundation for the use of SynComs technology to reveal the function of plant endophyte communities and the interaction mechanisms with plants. The SynCom construction process is regulated by multiple factors, such as the microorganisms themselves, plant hosts, and environment, among which the effects of microorganisms themselves include interspecies interactions, metabolism, and spatial structures [117]. Therefore, the construction of SynComs requires comprehensive consideration of the interactions among species, metabolism, and spatial structures to ensure the stability of SynComs.

### 4.2. Application of Omics to Elucidate the Mechanisms by Which Endophytes Promote the Occurrence and Accumulation of Plant Secondary Metabolites

**Figure 5:**
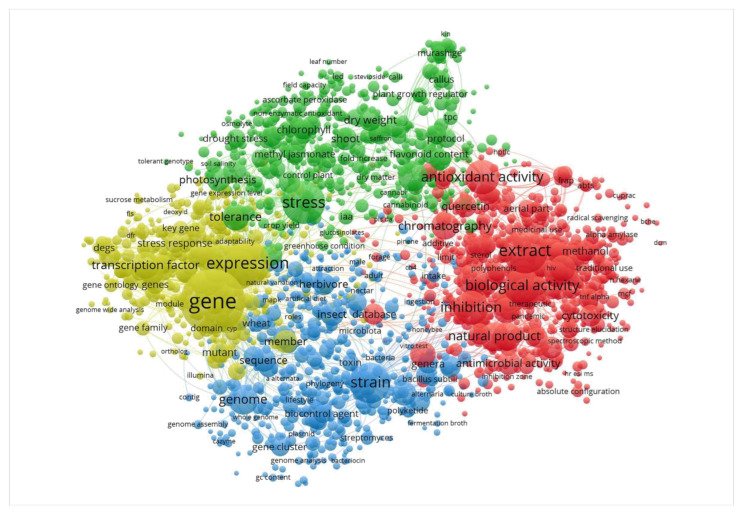
Visualization of the popular research topics related to plant secondary metabolites in the past five years. VOSviewer software was used to analyze the titles and abstracts of 12,745 articles in the Web of Science database from the past five years (1 January 2018–11 October 2022). The results showed that the current popular research topics mainly focus on three aspects of plant genes: type, stress, and metabolism. The retrieval conditions used in the database are (ALL = (Secondary metabolites)) AND ALL = (plant), the export format is a TAB delimited file, and the record content is “Full record and referenced references”. The lowest frequency of keywords screened by VOSviewer software was set as 15, and 4523 keywords were screened in total. Among 2714 keywords with correlations greater than or equal to 60%, 351 uninformative keywords were manually removed, and 2363 keywords were finally used for visual analysis.

Endophytes promote the synthesis of plant secondary metabolites in various complex ways, and traditional research methods have been insufficient for studying secondary metabolites through these relationships [118]. Studies on plant secondary metabolites have focused on their genetics, responses to stress, metabolism, and structures (Figure 5). Among existing studies and analyses, the main research methods used to explore the interactions between endophytes and plants include next-generation sequencing (NGS) and omics analysis. To date, NGS has been widely used to study the diversity of microbial communities in various environments [119,120,121]. In addition, this technology can also be used for large-scale genome sequencing [119,122], gene expression analysis [123,124], the identification of noncoding small RNAs [125,126], the screening of transcription factor target genes [127,128], and DNA methylation [129,130]. However, NGS has limitations, such as the possible detection of plant DNA when studying microbial communities interacting with plants, thereby reducing the amount of data acquired for microbes. Thus, to avoid interference with plant sequences, we can increase the specificity of the primers or improve the ability of high-throughput data acquisition to obtain additional information. With technological advancement, NGS will gradually improve. Currently, omics, as an effective complement to this technique, plays an essential role in the analysis of plant secondary metabolites, particularly the joint effect of multiomics as a directional indicator to study the interactions between endophytes and plants.

To date, the application of multiomic technology in studies on secondary metabolism to determine the interactions between plants and endophytes is becoming more widespread [128,131]. In research, multomics generally takes the form of metaomics, which mainly includes metagenomics, macrotranscriptomics, and macroproteomics. By combining metagenomic, macrotranscriptomic, and macroproteomic methods, metaomics can be used to not only predict the potential functions of endophytic communities, but also to determine the functional activity of endophytic communities [40,132]. At the same time, a more comprehensive understanding of endophytic communities can be promoted by determining the intraspecific relationships of communities, understanding nutrient competition between plants and endophytes, and examining community development [133,134]. In recent years, metaomics has been the focus and frontier of research in the field of plant–endophyte interactions, with remarkable results. With the development of whole-metagenome shotgun (WMS) sequencing technology, research using metaomics has changed from high-depth sequencing, which can only reflect the characteristics of one sample, to large-sample sequencing, which can reflect the differences between different samples. Studies have shifted from sample sequencing at one time point to dynamic process sequencing in response to different external environments [132,135].

However, the current metaomic research methods still have some shortcomings and inadequacies. Metaomic analysis of plant samples, while potentially capable of representing all the diversity in gene sequence and function, has not been able to elucidate all the genes and functions of a single microorganism, and this has presented great difficulties in exploring the functions of plant endophytes. Fortunately, modern methods such as single-cell genome sequencing (SCGS) have been slowly developed [136]. Therefore, we should look forward to the development of SCGS and alternative techniques to address these difficult issues in studying the interaction between plants and endophytes and to further elucidate the mechanisms by which endophytes facilitate the occurrence and accumulation of secondary metabolites in plants.

### 4.3. Endophytes Advance the Application of Plant SMs

Endophytes are ideal resources for facilitating the formation and accumulation of secondary metabolites in plants. Earth is a large biological resource bank, with approximately 270,000 plant species, and the number of endophytes that can be isolated exceeds one million based on the calculation that there are approximately four distinct endophytes in each plant [137]. The study of endophytes has opened up new areas for research into the application of microorganisms. The current work focuses on the use of screening to promote the formation and accumulation of plant secondary metabolites in host strains and the transformation of strains to improve the breeding process, industrialized production, and production capacity, as well as to improve the direct application of plant secondary metabolites in food, agriculture, and other areas [138]. In addition, various plant secondary metabolites have the potential to be indirectly exploited as biopesticides, pharmaceuticals, or pharmaceutical precursors, and these secondary metabolites possess a wide range of activities, including antimicrobial, antioxidant, and biocontrol activities [18,139]. At the same time, the interaction between endophytes and plants produces new bioactive secondary metabolites, providing feasible opportunities for the development of improved drugs and more effective ways for humans to cure diseases in the future [140]. This result clearly shows that using endophytes promotes the synthesis of host plant secondary metabolites and the generation of new active substances. This solves the issues with using traditional methods involving the long growth cycle and restrictions on the active ingredients needed to produce plant resources. This method is expected to provide a new way to quickly produce active substances in plants and will have great application value and development potential.

## 5. Expectation

Endophytes are an important part of the plant microecosystem. They have a special symbiotic and metabolic relationship with host plants and are a potential resource pool for the synthesis and accumulation of plant secondary metabolites. At present, research on endophytic bacteria in plants is still in its infancy, and only some plant groups have been studied. The existing research is more focused on endophytes as a resource, and their various novel structures and bioactive secondary metabolites have been discussed. As a result, most research on the substances in host plants has been confined to those that are the same or similar to active substances in endophytes. Thus, the construction of a systemic database of endophytes and plants is not possible due to the lack of information and the need for an endophyte resource platform. Endophytes play an important role in the development of plant secondary metabolites and in promoting the synthesis and accumulation of plant secondary metabolites. Many bioactive plant secondary metabolites are transformed by endophytes to induce plant synthesis and accumulation. However, the structure, dynamics, and functions of plant endophytic colonies, the factors influencing the interaction between plant secondary metabolites and endophytic colonies, the spatial patterns and signal transduction pathways, and the specific mechanism and molecular basis of the regulation of the biosynthesis of plant secondary metabolites by endophytes still need to be further explored. In addition, the fundamental genetic control of endophytes in plants and how they are affected by changes in other microbiota and environmental conditions also require attention.

In fact, although researchers have found valuable active substances in the study of some endophytes promoting plant secondary metabolites, many problems have yet to be addressed in related research and practical applications. Most plant endophytes have not been effectively isolated, and the molecular mechanisms and effects of plant endophytes are not clear. In addition, endophytes cannot be used in practice for biocontrol, and it is not clear what kind of community they need in different growth stages and when they act on plants. Therefore, further exploration of the interaction between endophytes and plants is urgently needed to understand the origin and mechanism of action of endophytes in plants.

Interdisciplinary research in plant physiology, molecular biology, genetics, microbiology, and many other disciplines has been formed in many studies. With the help of analytical tools such as single-cell genomics and specialized plant metabolomics, we can focus on identifying gene clusters of secondary metabolite biosynthetic pathways within plants through whole-genome sequences to investigate the resources of novel endophytes with sustainable secondary metabolite synthesis and to reveal the molecular signals and transcription factors that regulate the expression of secondary metabolic biosynthesis-related gene clusters. These data can be used to explore the microbial communities coevolving with plants as they grow and develop and to provide a new theoretical basis and research perspective for revealing the regulation of plant growth and development by endophytes through secondary metabolites and the industrialization of synthetic communities. In the future, in-depth research on plant endophytes and their applications in various fields will bring more benefits to humans and will have broad prospects for development and application.

## Figures and Tables

**Table 1:** Endophytes affect the accumulation of photosynthetic components in the host.

Microbial Classification	Endophytic Strains	Sources	Changes in Matter	Functions	Reference
Endophytic fungi	*Cystobasidiales* and, *Chaetothyriales*	*Ulmus minor Mill.*	Antioxidant enzymes and Auxin	Promote the growth to rhizome and adjust the resistance of Dutch elm disease (DED) disease in *Ulmus*.	[68]
	*Saccharomyces cerevisiae*, *Zygosaccharomyces bailii*, and *Saccharomyces kudriavzevii*.	*Tobacco*	(3-indoleacetic acid) IAA, and elevated chlorophyll levels.	Promote the growth and development of tobacco and disease resistance.	[69]
	*Alternaria sorghi* and, *Penicillium commune*	*Phaseolus vulgaris* L.	Photosynthetic pigments, carbohydrate, and protein contents.	Promote the growth and biomass accumulation of common legumes.	[70]
	*Penicillium brevicompactum* and, *P. chrysogenum*	*Tomato* and *Lettuce*	Nutrients and Na^+^ contents, net photosynthesis, water use efficiency.	Improve the survival rate and material accumulation of plants under adverse environment.	[71]
	*Arbuscular mycorrhizal* fungi and Dark septate endophytes (DSE)	*Reed*	Antioxidant enzymes	Improve the growth, nutrient content, and photosynthesis of *Phragmites* *australis*.	[72]
	*Gilmaniella* sp. AL12	*Atractylodes lancea*	Terpenoid	Improve photosynthesis and biomass synthesis.	[73]
	*Cercospora*	*Tricyrtis macropoda*	Amino acids, organic acids, lipids.	Improve photosynthesis and biomass synthesis.	[74]
Endophytic bacteria	*Enterobacter* sp. MN17	*Pisum sativum*	Protein, fat, fiber, and ash	Improve photosynthesis efficiency and cadmium (Cd) pollution.	[75]
	*Bacillus altitudinis* HNH7 and *Bacillus velezensis* HNH9	*Cotton*	IAA	Promote the growth of cotton plants	[76]
	*Bacillus megaterium* ZS-3	*Arabidopsis thaliana*	Chlorophyll (Chl) content and carotenoid content	Promote plant growth in salt-stressed environments.	[77]
	*Bacillus subtilis*	*Phaseolus vulgaris* L.	Chl a and Chl b, lignin.	Promote plant growth and stress tolerance.	[78]
	*Bacillus thuringiensis* and, *Brevibacillus agri*	*Phaseolus vulgaris* L.	Photosynthetic pigments, carbohydrate, and protein contents.	Promote the growth and biomass accumulation of common legumes.	[70]
	*Burkholderia*, *Pseudomonas* and, *Azospirillum*	No data	Shoot biomass, relative water content, sugar, and proline concentrations.	Improve the cold resistance of plants and regulate their growth.	[79]

**Table 2:** Endophytes affect the expression of plant-related genes.

Microbial Classification	Endophytic Strains	Sources	Differences in Gene Expression	Functions	Reference
Endophytic fungi	*Penicillium brevicompactum* and, *P. chrysogenum*	*Tomato* and *Lettuce*	Enhanced expression of the *NHX1* gene.	Improved sequestration of Na^+^ in vacuoles is suggested by the upregulation of the expression of vacuolar NHX1 Na^+^/H^+^ antiporters.	[71]
	*Gilmaniella* sp. AL12	*Atractylodes lancea*	Genes and proteins related to primary metabolism (carbon fixation, carbohydrate metabolism, and energy metabolism) tended to be upregulated.	Improved photosynthesis and biomass synthesis.	[73]
	*Paecilomyces variotii*	*Arabidopsis thaliana*	Enhanced the expression of IAA related genes.	The reactive ROS production promoted plant growth, yield, and quality parameters, and increased the absorption of nitrogen from the plant.	[82]
	*Biscogniauxia* sp. and *Didymella* sp.	*Vitis amurensis Rupr*	Activation of phenylalanine ammonialyase (*PAL*) and stilbene synthase (*STS*) gene expression.	Stimulated the production of stilbenes.	[83]
	*Serendipita indica* and *Serendipita herbamans*	*Solanum lycopersicum*	*SPS-A1* gene expression and *SWEET11b* expression.	Sucrose resynthesis in roots.	[84]
	*Pochonia chlamydosporia*	*Tomato*	Expression changes of *PAL*, *PIN II*, *PR1* and *LOX D.*	Activated plant defense response and improved survival ability.	[85]
Endophytic bacteria	*Bacillus altitudinis* HNH7 and *Bacillus velezensis* HNH9	*Cotton*	Upregulated the expression of growth-linked genes, *EXP6*, *ARF1*, *ARF18*, *IAA9*, *CKX6,* and *GID1b*, and downregulated *ERF* and *ERF17*.	Promoted the growth of cotton plants.	[76]
	*Enterobacter hormaechei* H2A3 and H5A2.	*Stevia rebaudiana*	The increase in the transcript levels of the *KO, KAH, UGT74G1*, and *UGT76G1* genes.	Increased steviol glycosides (SG) synthesis, flavonoid content, and flavonoid accumulation.	[86]
	*Pseudomonas*	*Cotton*	Downregulated *GhCAX3* expression.	Increased intracellular calcium ion (Ca^2+^), hydrogen peroxide (H_2_O_2_), and nitric oxide (NO) contents. The coordinated regulation of Ca^2+^, H_2_O_2_, and NO enhanced cotton resistance to Verticillium wilt.	[87]
	*Bacillus circulans* GN03	*Cotton*	Upregulated the expression of phytohormone synthesis-related genes (*EDS1*, *AOC1*, *BES1*, and *GA20ox*), auxin transporter gene (*Aux1)*, and disease-resistance genes (*NPR1* and *PR1*).	Enhanced growth promotion as well as disease resistance.	[88]

**Table 3:** Endophytes produce the same or similar secondary metabolites as the host plant.

Microbial Classification	Endophytic Strains	Sources	The Same or Similar Secondary Metabolites	Functions	Reference
Endophytic fungi	*Pyricularia oryza*	*Rice*	Melanin, nectriapyrones, tenuazonic acid.	It can effectively prevent plant diseases and pests.	[92]
	*Eupenicillium parvum*	*Azadirachta indica*	Azadirachtin A and B.	Having antifeedant and insect growth-regulating properties.	[93]
	*Pestalotiopsis fici*	*Camellia sinensis*	Polyketide, nonribosomal peptides, alkaloids, and terpenes.	Promotes the accumulation of plant active ingredients and plant growth.	[94]
	*T. asperellum*, *T. brevicompactum*, *T. koningiopsis*, and *T. longibrachiatum*	*Vinca major*, *Vinca herbacea*, and *Vinca minor*	Alcohols, esters, pyrones (lactones), acids, furanes, and lipids.	Antibacterial and cytotoxic activities and can promote plant growth.	[95]
Endophytic bacteria	Bacterial strain *Bvel1*	*Grape*	Iturin A2, surfactin-C13 and -C15, oxydifficidin, L-dihydroanticapsin, and azelaic acid.	Antifungal activity; promotes wound healing in plants.	[96]
	*Pseudomonas*, *Xanthomonas*, *Variovorax*, *Bacillus*, *Pantoea*, and *Stenotrophomonas*	*Alkanna tinctoria*	Pectinase and cellulase	It has antibacterial and antitumor properties, promoting wound healing and plant growth.	[97]
	*Acinetobacter baumannii*	*Capsicum annuum* L.	Phenols, carboxylic acids, aromatic heterocyclic compounds, ketones, aromatic esters, aromatic benzenes, and alkenes.	Antioxidation; promotes plant growth.	[98]
	*Bacillus atrophaeus* and *Bacillus mojavensis*	*Glycyrrhiza uralensis*	1,2-benzenedicarboxylic acid, bis (2-methylpropyl) ester; methyl ester; 9-octadecenoic acid,; and decanedioic acid, bis(2-ethylhexyl) ester.	Resistance to Verticillium wilt disease and other phytopathogens.	[99]
	*Pseudomonas fluorescens* ALEB7B	*Atractylodes lancea*	IAA	It triggers the oxidation burst, stimulates the conversion of terpene hydrocarbon scaffolds into active components containing oxygen sesquiterpenes, and promotes the accumulation of plant biomass and plant growth.	[100]
	*Microbacterium* and *Burkholderia*	*Coptis teeta*	Berberine	Enhances plant nutrient absorption, promotes growth, prevents pathogen damage.	[101]
	*Bacillus subtilis* LB5	*Chuanxiong Rhizoma*	Ligustrazine	Promotes plant growth and biomass synthesis. Simultaneously produced metabolites can treat ischemic vascular-related diseases.	[102]

## Data Availability

No new data were created or analyzed in this study. Data sharing is not applicable to this article.
